# Multidisciplinary Vulvar Cancer Management: The Dermatologist’s Perspective

**DOI:** 10.3390/life15010019

**Published:** 2024-12-27

**Authors:** Marta Cebolla-Verdugo, Victor Alfredo Cassini-Gómez de Cádiz, Juan Pablo Velasco-Amador, María Zulaika-Lloret, Francisco Manuel Almazán-Fernández, Ricardo Ruiz-Villaverde

**Affiliations:** 1 Department of Dermatology, Hospital Universitario San Cecilio, Avenida del Conocimiento s/n, 18016 Granada, Spain; elromeral28a@gmail.com (V.A.C.-G.d.C.); pablova.correo@gmail.com (J.P.V.-A.); zulaikalloretmaria@gmail.com (M.Z.-L.); almazanweb@gmail.com (F.M.A.-F.); ismenios2005@gmail.com (R.R.-V.); 2 Instituto Biosanitario de Granada, Ibs, 18012 Granada, Spain

**Keywords:** vulvar cancer, squamous cell carcinoma (SCC), vulvar melanoma, dermatologist, radical vulvectomy, postoperative care, wound management, multidisciplinary approach, targeted therapies

## Abstract

Vulvar cancer, particularly squamous cell carcinoma (SCC) and melanoma, poses significant diagnostic and therapeutic challenges due to its complex presentation and high rates of postoperative complications. Effective management requires a multidisciplinary approach, integrating the expertise of gynecologic oncologists, dermatologists, plastic surgeons, and other specialists. This review highlights the dermatologist’s role in supporting early diagnosis, addressing predisposing conditions such as lichen sclerosus, and managing postoperative wound complications, including surgical site infections and dehiscence. Through two clinical cases, we illustrate how dermatological expertise complements surgical efforts by employing advanced wound care techniques such as negative pressure wound therapy and tailored dressing protocols. By collaborating closely with gynecologists and other team members, dermatologists enhance patient outcomes, ensuring timely recovery and the prevention of long-term sequelae. The article also discusses recent advances in treatment guidelines and targeted therapies, underscoring the importance of integrated care for optimizing patient outcomes in vulvar cancer.

## 1. Introduction

Vulvar cancer accounts for approximately 5% of all gynecological malignancies, with squamous cell carcinoma (SCC) representing 90–95% of cases, and melanoma comprising 2–4% [1]. In recent years, the incidence of vulvar cancer has been increasing, particularly among younger women, largely due to the growing prevalence of human papillomavirus (HPV) infections [2]. HPV-related SCC typically affects younger women, while non-HPV-related SCC is more common in older women and is often associated with chronic inflammatory dermatoses, such as lichen sclerosus, which can predispose patients to malignant transformation.

The management of vulvar cancer is complex, requiring a comprehensive approach that integratesmultiple specialties. Collaboration between dermatologists, gynecologic oncologists, radiation oncologists, plastic surgeons, psychologists, and wound care specialists is essential to address the diverse challenges of this disease. Dermatologists contribute significantly by aiding in the early diagnosis of vulvar lesions, managing chronic inflammatory conditions that predispose to malignancy, and providing specialized care for postoperative complications, such as wound infections and dehiscence. Meanwhile, oncologists and radiation oncologists play key roles in systemic and adjuvant therapies, complementing surgical interventions provided by gynecologic and plastic surgeons [3].

This review explores the role of dermatologists as integral members of the multidisciplinary team managing vulvar cancer. By focusing on their contributions to diagnosis, postoperative care, and collaboration with other specialists, as well as presenting case-based examples, this article aims to offer insights into optimizing outcomes for patients with vulvar malignancies.

Dermatologists are integral members of multidisciplinary teams managing vulvar cancer, contributing at various stages from diagnosis to postoperative care. Their expertise in skin and mucosal diseases makes them particularly adept at addressing both malignant and non-malignant conditions associated with vulvar pathology.

## 2. Early Diagnosis and Management of Chronic Dermatoses

Dermatologists are often the first specialists to identify suspicious vulvar lesions, initiating diagnostic procedures such as punch or excisional biopsies and ensuring timely referral to gynecologic oncologists. This early intervention is crucial for optimizing patient outcomes.

Dermatologists are often the first specialists to identify suspicious vulvar lesions, performing biopsies and ensuring timely referral to gynecologic oncologists, which is crucial for optimizing patient outcomes. They also manage chronic inflammatory conditions like lichen sclerosus and lichen planus, which increase the risk of vulvar squamous cell carcinoma. A notable example is fractional CO_2_ laser therapy for lichen sclerosus unresponsive to corticosteroids, demonstrating efficacy in alleviating symptoms and improving quality of life [4,5,6,7,8]. The International Society for the Study of Vulvar Disease (ISSVD) recognizes dermatologists’ expertise as essential for comprehensive vulvovaginal care [9].

## 3. Postoperative Wound Care and Complication Management

Rates of complications following radical vulvectomy range from 20% to 60%, with factors such as surgical site infections, wound dehiscence, and lymphedema contributing to patient morbidity [10]. In cases where inguinofemoral lymph node dissection (IFLND) is performed, complication rates can rise to as high as 85%, with dehiscence occurring in 70–90% of cases. Dermatologists contribute to managing these complications by employing advanced wound care strategies, including moisture-retentive dressings and negative pressure wound therapy (NPWT), which promote healing and reduce the risk of long-term sequelae. Their expertise ensures that patients receive individualized and effective care during the challenging postoperative period [11].

## 4. Multidisciplinary Collaboration

The complexity of vulvar cancer necessitates a multidisciplinary approach, with dermatologists collaborating closely with gynecologic oncologists, radiation oncologists, plastic surgeons, psychologists, and wound care specialists. Dermatologists assist in diagnostic processes, guide the management of chronic vulvar conditions, and contribute to postoperative care. Their role extends to working with plastic surgeons to address scarring and chronic wounds after reconstructive surgery and collaborating with radiation oncologists to monitor and manage dermatological side effects of adjuvant therapies. Additionally, they partner with psychologists to address the significant emotional and psychosocial impact of vulvar cancer, supporting patients through challenges such as anxiety, depression, and sexual dysfunction. This collaborative framework underscores the vital role of dermatologists in optimizing both clinical and psychosocial outcomes for patients.

## 5. Squamous Cell Carcinoma of the Vulva

Vulvar squamous cell carcinoma (SCC) is a type of cancer originating in the squamous cells of the vulva, accounting for more than 90% of malignant vulvar tumors [10]. It is most diagnosed in postmenopausal women, with a median age at diagnosis of 70 years, although the incidence has been rising among younger women due to an increased prevalence of human papillomavirus (HPV) infections [12]. Vulvar SCC can develop from precursor vulvar intraepithelial lesions, known as vulvar intraepithelial neoplasia (VIN), which may precede invasive carcinoma for a variable period [13].

### 5.1. Risk Factors

Several risk factors contribute to the development of vulvar SCC:

- **Human papillomavirus (HPV) infection:** HPV infection, particularly with high-risk types such as HPV-16 and HPV-18, is a major risk factor. Vulvar SCC can develop through two distinct pathways: one associated with persistent HPV infection and another independent of HPV [11,12]. HPV-related cases are more common in younger women, while HPV-independent SCC often occurs in older women.
- **Smoking**: Tobacco use is strongly associated with an increased risk of vulvar SCC, particularly in cases related to HPV infection [14,15].
- **Chronic inflammatory conditions**: Conditions such as lichen sclerosus and lichen planus increase the risk of developing vulvar SCC, especially through the HPV-independent pathway. Chronic inflammation and scarring can lead to malignant transformation over time [10,11]. Early recognition and effective management of these conditions, including advanced interventions such as fractional CO_2_ laser therapy (as discussed earlier) [4,5,6,7,8], are critical to reducing cancer risk and improving patient outcomes.
- **Socioeconomic factors:** Variables such as marital status and educational level have also been associated with an increased risk of vulvar SCC [14].
- **History of cervical intraepithelial neoplasia (CIN):** Women with a history of CIN have a higher risk of developing vulvar SCC [16].
- **Obesity**: Obesity is an additional risk factor, especially for HPV-unrelated vulvar SCC [17].

In summary, vulvar SCC is a malignancy that can develop from precursor lesions and is associated with several risk factors, including HPV infection, smoking, chronic inflammatory conditions, socioeconomic factors, history of CIN, and obesity.

### 5.2. Management of Vulvar SCC

The management of vulvar SCC follows a well-defined protocol that varies according to the stage of the disease and the characteristics of the tumor. It involves a combination of surgical and non-surgical treatments aimed at achieving locoregional control while minimizing morbidity.

### 5.3. Surgical Management

For early-stage disease (IA and IB), the primary treatment is surgical excision with appropriate margins of 1–2 cm, ensuring the removal of the tumor while preserving surrounding healthy tissue [18,19]. Evaluation of the inguinofemoral lymph nodes (IFLNs) is critical to determine the extent of disease spread. Sentinel lymph node biopsy (SLNB) is preferred to reduce morbidity compared to complete lymphadenectomy, particularly in early-stage cases without clinically evident lymph node involvement [19].

### 5.4. Advanced Disease Management

In cases of locally advanced disease (stages II–IVa), a multimodal approach is recommended. Primary chemoradiation is a viable option, especially for tumors that are not amenable to surgical resection. A phase II study demonstrated that chemoradiation with capecitabine provides effective locoregional control with acceptable survival rates [18,20]. Alternatively, neoadjuvant chemotherapy followed by surgery is an evolving strategy, aiming to shrink tumors before surgical removal to improve locoregional control and preserve quality of life [20].

### 5.5. Adjuvant Therapy

For patients with nodal metastases or additional risk factors such as positive surgical margins, lymphovascular invasion, or nodal metastasis, adjuvant radiotherapy is recommended following surgery [18]. Postoperative radiotherapy plays a crucial role in reducing the risk of local recurrence and improving overall survival in high-risk patients [21,22].

In conclusion, the management of vulvar SCC is guided by the stage of the disease and the specific characteristics of each case. A multidisciplinary approach involving gynecologic oncologists, dermatologists, and radiation oncologists is essential to tailor treatment strategies and optimize patient outcomes.

## 6. Vulvar Melanoma

Vulvar melanoma is a rare type of cancer that originates in the pigment-producing cells (melanocytes) of the vulva. It accounts for approximately 5% of all vulvar neoplasms and is the second most common type of vulvar cancer after squamous cell carcinoma [23,24].

### 6.1. Risk Factors

- **Advanced age**: Vulvar melanoma primarily affects older women, with a higher incidence among elderly Caucasian women [23,25].
- **Chronic inflammatory Diseases**: Conditions such as lichen sclerosus and lichen planus increase the risk of developing vulvar melanoma [26,27].
- **Viral infections**: Although not fully defined, viral infections are considered a potential risk factor for vulvar melanoma [26,27].
- **Irritant exposure**: Chronic exposure to irritants may contribute to the development of vulvar melanoma [26,27].
- **Genetic susceptibility**: Mutations in genes such as KIT, BRAF, and NRAS are common in vulvar melanoma and may serve as therapeutic targets [23].

Unlike cutaneous melanoma, vulvar melanoma is not associated with ultraviolet radiation exposure, as it develops in an area that is protected from the sun [26,27]. Early detection and appropriate treatment are critical due to the high recurrence rate and metastatic potential of this type of melanoma [23,24].

### 6.2. Management Protocol for Vulvar Melanoma

The management of vulvar melanoma involves several stages and therapeutic options, depending on the stage of the disease at diagnosis.

- **Localized disease (stages I and II)**: The standard treatment is wide surgical excision with appropriate margins. Surgery may include radical vulvectomy or wide local excision, depending on the tumor’s thickness and extent. Sentinel lymph node biopsy is recommended to evaluate lymphatic spread and to avoid the morbidity associated with complete lymphadenectomy [28,29].
- **Advanced disease (stages III and IV)**: A multimodal approach is required for advanced cases. Therapeutic options include:
  1. **Immunotherapy**: Immune checkpoint inhibitors, such as nivolumab and ipilimumab, have shown efficacy in treating metastatic vulvar melanoma. A retrospective study reported an objective response rate of 33.3%, with a safety profile like that of cutaneous melanoma [30,31].
  2. **Targeted therapy**: In cases with specific mutations, such as BRAF or KIT, tyrosine kinase inhibitors can be considered. These treatments have demonstrated clinical efficacy in metastatic vulvar melanomas with specific mutations [28,30].
  3. **Chemotherapy**: Although chemotherapy has not shown a clear survival benefit, it can be used in combination with other treatments. The combination of carboplatin and paclitaxel with bevacizumab has shown promising results in some cases [29].
  4. **Radiotherapy**: Adjuvant radiotherapy may be used in cases with positive margins or nodal metastasis, although its benefit in improving survival is not clearly established [28].

## 7. Role of Targeted Therapies

The introduction of immunotherapies and targeted therapies, particularly for advanced vulvar melanoma, has significantly expanded the treatment landscape. Immune checkpoint inhibitors and targeted therapies like BRAF inhibitors offer new hope for patients with specific mutations, providing options beyond traditional chemotherapy.

1. **BRAF mutations**: For melanomas with BRAF mutations, BRAF inhibitors like vemurafenib and dabrafenib, combined with MEK inhibitors like trametinib, have demonstrated significant survival benefits. These treatments are effective because they block the hyperactivated MAPK pathway in BRAF-mutated melanomas [32,33].
2. **KIT mutations**: For melanomas with KIT mutations, tyrosine kinase inhibitors such as imatinib and nilotinib are viable therapeutic options. Although the results are not as pronounced as those with BRAF and MEK inhibitors, these treatments have shown efficacy in melanomas with KIT mutations [34,35].

## 8. Prognosis

1. The prognosis of vulvar melanoma varies significantly depending on the stage at the time of diagnosis.
2. **Localized disease (stages I and II)**: Patients with localized disease have a relatively better prognosis. One study reported a 5-year disease-specific survival rate of 63.6% for these stages [36]. Another study indicated that the median survival for stage I is 72 months, while for stage II, it is 45 months [37].
3. **Regional disease (stage III)**: In contrast, patients with regional disease have a significantly worse prognosis. The 5-year disease-specific survival rate for stage III is 0% [38], with a median survival of 24 months [37]
4. **Metastatic disease (stage IV)**: For those with metastatic disease, the prognosis is very poor. The 5-year disease-specific survival rate is 22.1% [39], and the median survival for stage IV is 23 months [37].

In summary, the prognosis of vulvar melanoma heavily depends on the stage at diagnosis, with significantly better survival rates in early stages (I and II) compared to advanced stages (III and IV). The role of the dermatologist is important in the surgical approach of the first two stages and in the joint management of targeted therapy and control of possible dermatological adverse effects associated with it.

## 9. Surgical Considerations and Reconstructive Surgery

Surgical resection remains the cornerstone of treatment for both vulvar SCC and melanoma. Depending on the extent of the disease, surgeries range from local excisions to radical vulvectomy. Radical vulvectomy, which involves the removal of the entire vulva, may be necessary for large or advanced tumors, but it is associated with significant morbidity, including sexual dysfunction, chronic pain, and psychological distress [10]. Efforts are increasingly focused on limiting the extent of surgery to preserve function and quality of life. Less radical surgical techniques, such as wide local excision combined with SLNB, have been shown to provide excellent oncological outcomes with fewer complications [10].

In reconstructive surgery for vulvar cancer, several key factors must be considered to optimize outcomes and minimize complications:

1. **Defect assessment**: The geometry and size of the post-surgical defect are crucial in determining the appropriate reconstructive technique. It is essential to assess the extent of the defect and the availability of local tissues for reconstruction [40,41].
2. **Flap selection**: The choice of flap should be based on the location and size of the defect, as well as the availability of tissues following primary surgery. Commonly used flaps include the vertical rectus abdominis myocutaneous (VRAM) flap, the anterolateral thigh (ALT) flap, the V–Y fasciocutaneous flap, and the deep inferior epigastric perforator (DIEP) flap [40,41,42].
3. **Previous complications**: The presence of scars, prior incisions, and previous radiotherapy treatments can complicate the reconstruction process. It is crucial to consider these factors to appropriately plan the surgery and select the most suitable flap [40].
4. **Infection management**: Contamination by urinary and fecal pathogens in dehiscent wounds or ulcerated tumors is a common complication. Antibiotic prophylaxis and proper wound management are essential to prevent postoperative infections [40,41].
5. **Overall patient condition**: Factors such as age, body mass index (BMI), and comorbidities like diabetes can influence wound healing and the risk of complications. These factors must be considered when planning reconstruction [42].
6. **Functional and aesthetic outcomes**: The goal of reconstruction is not only to cover the defect but also to restore the functional and aesthetic appearance of the vulva. This is critical for improving the patient’s quality of life [42,43].

To sum up, dermatologists play a significant role in reconstructive surgery following vulvar cancer treatment, contributing to both the planning and postoperative management to ensure optimal outcomes. Dermatologists are particularly skilled in assessing the geometry and extent of post-surgical defects, considering factors such as local tissue availability, previous surgical scars, and radiation damage. Their expertise is critical in flap selection, helping to determine the most appropriate reconstructive technique, whether using local flaps like the V–Y fasciocutaneous flap or more complex options such as the vertical rectus abdominis myocutaneous (VRAM) flap or the anterolateral thigh (ALT) flap. Dermatologists also address common complications, including infection management in contaminated wounds, through targeted antibiotic prophylaxis and advanced wound care strategies. Beyond defect coverage, dermatologists prioritize restoring the functional and aesthetic integrity of the vulva, focusing on outcomes that enhance the patient’s quality of life, including sexual function and psychological well-being. This multidisciplinary collaboration highlights the dermatologist’s essential role in improving both surgical and long-term patient-centered outcomes in vulvar cancer care.

## 10. Complications Associated with Vulvar Cancer Treatment

Vulvar cancer treatments are associated with several common complications, both in the short and long term. These complications vary depending on the type and extent of treatment, particularly with surgery and radiotherapy.

### 10.1. Surgical Complications

1. **Wound dehiscence**: Vulvar wound dehiscence is a frequent complication, reported in up to 18.1% of cases [44].
2. **Wound infection:** Wound infections are common, with rates ranging from 13.7% to 23.2% [45,46].
3. **Lymphoceles**: Lymphocele formation occurs in approximately 17.7% of patients [44].
4. **Lymphedema**: Persistent lymphedema is a significant complication, especially after inguinofemoral lymphadenectomy (IFL), with rates of up to 38.6% [45,46].

### 10.2. Radiotherapy Complications

1. **Cutaneous and mucosal adverse rvents:** Radiotherapy can cause acute and late skin and mucosal adverse events, with an incidence of grade 3 or higher toxicity reported in 54% of cases [18].
2. **Fibrosis and stenosis:** Vulvovaginal fibrosis and stenosis are long-term complications of radiotherapy [18].
3. **Osteoradionecrosis**: Radiotherapy may also predispose patients to osteoradionecrosis and stress fractures [18].

### 10.3. Risk Factors

Obesity and advanced age are independent risk factors for early complications, such as infections and wound dehiscence [45]. Diabetes and en bloc surgery also increase the risk of short-term complications [47].

### 10.4. Management of Surgical Complications

Managing surgical complications in vulvar cancer treatment, such as wound dehiscence, infections, lymphoceles, and lymphedema, requires a multidisciplinary approach and specific strategies tailored to each type of complication.

1. **Wound dehiscence:** Wound dehiscence is a common complication following vulvar surgery, with strategies available to reduce its incidence. The use of subcuticular sutures instead of staples has been shown to lower the risk of dehiscence, particularly when combined with adhesives like 2-octyl cyanoacrylate, which can also reduce prolonged wound drainage and shorten hospital stays [48,49]. Effective postoperative monitoring [50] is crucial for early detection of complications such as infections, seromas, or hematomas, allowing timely interventions like debridement to promote healing. Advanced moisture-retentive dressings [50] are beneficial, providing an optimal healing environment and reducing recovery time compared to traditional gauze dressings. Additionally, negative pressure wound therapy (NPWT) supports wound healing by enhancing blood flow, reducing bacterial burden, and encouraging granulation tissue formation, proving effective in complex cases [51]. Addressing patient-specific risk factors, such as controlling diabetes and promoting smoking cessation, is essential to further minimize the risk of dehiscence and improve overall surgical outcomes.
2. **Infections**: Wound infections are frequent and can be prevented using less radical surgical techniques, such as modified vulvectomy instead of en bloc radical vulvectomy. Antimicrobial prophylaxis does not always prevent infections, so proper wound management and close postoperative monitoring are crucial [52].
3. **Lymphoceles**: The formation of lymphoceles can be managed through careful monitoring and appropriate drainage. Prolonged use of suction drains can increase the risk of lymphedema, so balancing the duration of drainage is recommended. The use of sentinel lymph node biopsy can also reduce the incidence of lymphoceles compared to complete inguinofemoral lymphadenectomy [47,48].
4. **Lymphedema**: Lymphedema is a long-term complication that can be managed through early referral to specialized lymphedema services. Preserving the saphenous vein during lymphadenectomy and adopting less invasive surgical techniques, such as sentinel lymph node biopsy, can significantly reduce the incidence of lymphedema [53].

Dermatologists are key in managing complications from vulvar cancer surgery, including wound dehiscence, infections, lymphoceles, and lymphedema. They utilize advanced wound care techniques, such as negative pressure wound therapy, and promote less invasive approaches like sentinel lymph node biopsy to minimize risks. Through multidisciplinary collaboration, dermatologists ensure effective prevention, treatment, and improved patient outcomes.

## 11. Psychosocial Impact and Quality of Life

Vulvar cancer and its treatment have a profound impact on the psychosocial well-being of patients. Women diagnosed and treated for vulvar cancer face an increased risk of psychological distress, sexual dysfunction, and challenges in their intimate relationships [54,55]. Many patients experience symptoms of depression and anxiety, both before and after treatment. A longitudinal study found that 42% of women reported high levels of anxiety at diagnosis, which decreased to 30% during the first year of follow-up. Persistent vulvar symptoms, insomnia, and unmet informational needs are significantly associated with elevated anxiety levels [56]. Surgical treatments, such as vulvectomy, can negatively affect sexual function due to anatomical and physiological changes. Patients often report sexual dysfunction, including dyspareunia, abdominal pain during intercourse, and reduced ability to achieve orgasm, with such issues being common and persistent according to literature reviews [55,57]. The quality of intimate relationships is also impacted, with many women reporting lower satisfaction in their relationships and poorer quality of life compared to healthy women. Surgery and adjuvant treatments can exacerbate these challenges, affecting intimacy and communication with partners [57].

Dermatologists play a crucial role in identifying and addressing alterations in quality of life resulting from vulvar cancer and its treatments, such as surgery and radiotherapy. These treatments often lead to fatigue, pain, and impairments in physical, social, and sexual functioning. By closely monitoring patients, dermatologists can detect these issues early and collaborate with other specialists to implement comprehensive interventions, including physical therapy, pelvic floor therapy, sexual counseling, and psychotherapy, as recommended by the National Comprehensive Cancer Network (NCCN). Their involvement is integral to a multidisciplinary approach that mitigates the psychosocial impact of vulvar cancer, enhancing mental health, sexual function, and overall quality of life [18,58].

## 12. Case Reports: Lessons from Clinical Practice

To illustrate the role of dermatologists within the multidisciplinary management of vulvar cancer, we present two clinical cases. These cases highlight the dermatologist’s involvement in early diagnosis, management of chronic inflammatory conditions that predispose to malignancy, and the postoperative care of surgical complications. The following examples underscore the importance of individualized strategies and collaborative care in optimizing outcomes for patients with vulvar malignancies.

**Figure 1:**
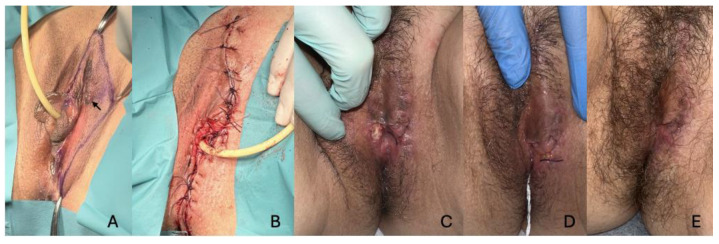
Evolution of the case of the patient with vulvar melanoma. (**A**). The image indicates with an arrow the presence of the nodule at the left vulvar level. Preoperative photo. (**B**). Photo of postoperative left hemivulvectomy. Monofilaments were applied afterward. (**C**). Slight wound dehiscence at the excision site of the contralateral minor labia. (**D**). Review at 2 weeks. (**E**). Appearance at 2 months.

The first case involves a 54-year-old woman who presented to the Dermatology department with a pruritic nodular lesion on the vulva of 1-year duration. Upon examination, she exhibited multiple hyperpigmented macules and a 1 cm nodule on the left vulva. Biopsies confirmed the presence of vulvar melanoma, leading to a left hemivulvectomy performed by the Gynecology service, along with a selective sentinel lymph node biopsy. Surgery achieved clear margins, and the sentinel lymph node biopsy was negative. During her hospitalization, meticulous wound care, including chlorhexidine sponge dressing changes, fibrin and eschar debridement, and the use of zinc sulfate-impregnated gauze, was employed to promote healing. The patient experienced slight wound dehiscence at the excision site of the contralateral minor labia but had otherwise uneventful wound epithelialization and was discharged after a week (Figure 1).

**Figure 2:**
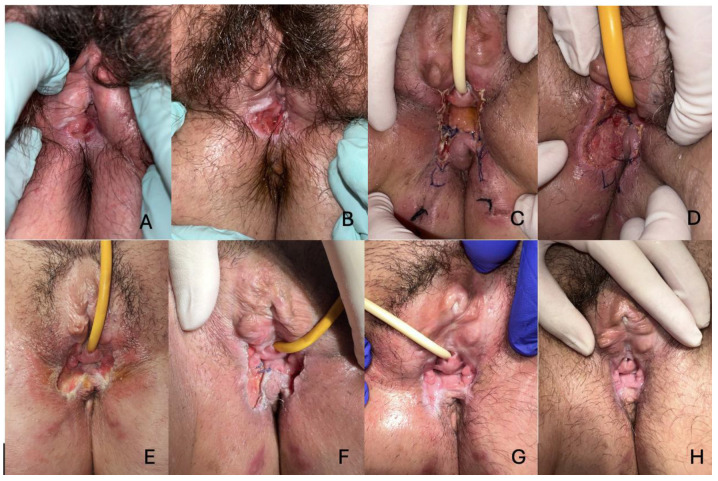
Evolution of the case of the patient with vulvar squamous cell carcinoma. (**A**). Ulcerated papule at the level of the right lower vulvar fossa. (**B**). Lesion extension over a month while the patient was on the surgical waiting list. (**C**). Inferior hemivulvectomy. Postoperative appearance. (**D**). Surgical wound dehiscence. (**E**). Appearance after daily wound care for a month. (**F**). Appearance at one and a half months. (**G**). Appearance at 2 months. (**H**). Current appearance.

The second case involves a 56-year-old woman with a 15-year history of lichen sclerosus who presented with a slightly exophytic, ulcerated nodule at the right inferior vulvar fossa. Biopsy revealed moderately differentiated squamous cell carcinoma. The patient underwent inferior hemivulvectomy. Despite wound care during her hospitalization, she experienced surgical wound dehiscence, requiring continued outpatient care at the Dermatology clinic. A healing ointment was applied to facilitate secondary intention closure, resulting in complete resolution of the wound. Additionally, fractional CO2 laser therapy was employed postoperatively to address residual scarring and pruritus associated with her lichen sclerosus. This treatment not only improved the appearance of surgical scars but also significantly alleviated her chronic symptoms, enhancing her overall quality of life (Figure 2).

These cases exemplify the critical importance of multidisciplinary collaboration in vulvar cancer management, with dermatologists contributing significantly to the integration of diagnostic, therapeutic, and postoperative strategies to optimize patient outcomes and quality of life.

## 13. Conclusions

The management of vulvar cancers, particularly squamous cell carcinoma (SCC) and melanoma, demands a comprehensive and multidisciplinary approach to address the complexities of diagnosis, treatment, and long-term care. Dermatologists play an essential role within this collaborative framework, contributing to the early detection of malignancies through biopsies, the management of chronic inflammatory conditions like lichen sclerosus that predispose to cancer, and the provision of advanced postoperative wound care for complications such as wound dehiscence and lymphedema. These efforts are enhanced by innovations such as fractional CO_2_ laser therapy, which exemplifies how dermatologists integrate cutting-edge treatments into care strategies [4].

Equally important is the collaboration between dermatologists, gynecologic oncologists, radiation oncologists, plastic surgeons, wound care specialists, and psychologists, among others. This interdisciplinary approach ensures that the physical, functional, and emotional needs of patients are addressed, from diagnosis to recovery. The inclusion of mental health professionals underscores the significance of supporting patients in coping with the psychosocial challenges posed by vulvar cancer.

Advances in less radical surgical techniques, immunotherapies, and personalized care strategies continue to improve outcomes. Ultimately, the integration of patient-centered care and seamless collaboration among specialists is critical to achieving optimal clinical and quality-of-life outcomes for individuals affected by vulvar cancer.

## Data Availability

The raw data supporting the conclusions of this article will be made available by the authors on request.
